# Proteome alteration induced by hTERT transfection of human fibroblast cells

**DOI:** 10.1186/1477-5956-6-12

**Published:** 2008-04-17

**Authors:** Gabriel D Mazzucchelli, Valérie Gabelica, Nicolas Smargiasso, Maximilien Fléron, Wilson Ashimwe, Frédéric Rosu, Marie-Claire De Pauw-Gillet, Jean-François Riou, Edwin De Pauw

**Affiliations:** 1 Laboratory of Mass Spectrometry; CART, GIGA, University of Liège, BAT. B6C, allée de la Chimie, 3, 4000 Liège 1, Belgium; 2 Histology and Cytology Laboratory, GIGA, University of Liège, BAT. B6C, allée de la Chimie, 3, 4000 Liège 1, Belgium; 3 Regulation et Dynamique des Genomes, Museum National d'Histoire Naturelle, USM 503, INSERM U565, CNRS UMR 5153, Paris, France

## Abstract

**Background:**

Telomerase confers cellular immortality by elongating telomeres, thereby circumventing the Hayflick limit. Extended-life-span cells have been generated by transfection with the human telomerase reverse transcriptase (hTERT) gene. hTERT transfected cell lines may be of outstanding interest to monitor the effect of drugs targeting the telomerase activity. The incidence of hTERT gene transfection at the proteome level is a prerequisite to that purpose. The effect of the transfection has been studied on the proteome of human fibroblast (WI38). Cytosolic and nuclear fractions of WI38 cells, empty vector transfected WI38 (WI38-HPV) and hTERT WI38 cells were submitted to a 2D-DIGE (Two-Dimensional Differential In-Gel Electrophoresis) analysis. Only spots that had a similar abundance in WI38 and WI38-HPV, but were differentially expressed in WI38 hTERT were selected for MS identification. This method directly points to the proteins linked with the hTERT expression. Number of false positive differentially expressed proteins has been excluded by using control WI38-HPV cells. The proteome alteration induced by hTERT WI38 transfection should be taken into account in subsequent use of the cell line for anti-telomerase drugs evaluation.

**Results:**

2D-DIGE experiment shows that 57 spots out of 2246 are significantly differentially expressed in the cytosolic fraction due to hTERT transfection, and 38 were confidently identified. In the nuclear fraction, 44 spots out of 2172 were selected in the differential proteome analysis, and 14 were identified. The results show that, in addition to elongating telomeres, hTERT gene transfection has other physiological roles, among which an enhanced ER capacity and a potent cell protection against apoptosis.

**Conclusion:**

We show that the methodology reduces the complexity of the proteome analysis and highlights proteins implicated in other processes than telomere elongation. hTERT induced proteome changes suggest that telomerase expression enhances natural cell repair mechanisms and stress resistance probably required for long term resistance of immortalized cells. Thus, hTERT transfected cells can not be only consider as an immortal equivalent to parental cells but also as cells which are over-resistant to stresses. These findings are the prerequisite for any larger proteomics aiming to evaluate anti-telomerase drugs proteome alteration and thus therapeutics induced cell reactions.

## Background

Telomeres are specialized functional DNA-protein complexes that cap the end of linear chromosomes. Their role is to protect chromosomes from degradation, recombination, or fusion, and to prevent the chromosome ends from being detected as strand breaks. At each cell division, telomeres shorten until they reach a critical size that drives eukaryotic cells into replicative senescence. Telomere length therefore acts as a biological life clock. Telomerase, a ribonucleoprotein complex, is involved in telomere length maintenance in eukaryotic cells by adding telomeric repeats to the 3' end of chromosomes. Telomerase activity is downregulated in most human cells during embryogenesis, thereby limiting their proliferative capacity. However, the reactivation of telomerase activity is observed in 90% of all human tumor cells, making this enzyme an attractive target for selective cancer therapy.

Reconstitution of telomerase activity by ectopic expression of the catalytic telomerase subunit (hTERT) cDNA stabilizes the telomere length of fibroblasts and other cell types that therefore acquire immortality [1]. Such hTERT-transfected cells have been proposed as immortal versions of normal human cells model with the advantage of indefinite proliferation. This strategy is applied for biochemical and physiological studies of normal cell growth, differentiation, genetic manipulation, etc [1-4]. More recently, experiments using transplanted telomerase-immortalized cells have been conducted in immunodeficient mice [5]. Adenocortical hTERT immortalized cells were able to replace cells with deficient functions. However such cell-based therapies raise important interrogations about medium and long term incidence of hTERT-immortalized cell autotransplantation cells. Indeed, several studies have evidenced that hTERT has other physiological roles beyond maintaining telomere such as potent incidence on malignant transformation of human fibroblasts by a telomere length-independent mechanisms [6-8]. Furthermore, the key role of telomerase in cancer cell immortalization led to the development of therapeutic strategies based on telomerase inhibition. A detailed characterization of hTERT-related transfection processes is therefore of outstanding interest to monitor anti-telomerase drug therapy.

We have studied here the sub-proteome modifications induced by hTERT transfection in the normal human fibroblast WI38 cell line. Cytoplasmic and nuclear fractions were analyzed by 2D DIGE. Comparison with controls, WI38 transfected with the empty vector (HPV) or parental untransfected WI38 cells has revealed the altered expression of proteins involved in apoptosis, cell cycle and endoplasmic reticulum homeostasis. The subfractionation method used allows telomerase detection by western blots, TRAP assay (Telomere repeat amplification protocol) experiment and reproducible extraction and isolation of the cytosolic and nuclear sub-proteomes, the latter one being enriched in low abundant proteins such as transcription factors and telomeric proteins. The interest of this work is to characterize the effect of hTERT expression at the proteome level of WI 38 cells for further investigating the effect of anti-telomerase drugs therapies in an integrated proteomic study.

## Results and Discussion

To characterize the protein expression changes resulting from hTERT transfection, we performed a differential proteomic analysis in order to compare wild type WI 38 cells (WI38), hTERT transfected WI 38 cells (WI38-hTERT) and control HPV transfected WI 38 cells (WI38-HPV) harvested at the same number of population doublings (PDL) after transfection and FACS sorting (PDL = 10). The use of this relatively low passage number *in vitro *maintains in hTERT transfected cells normal cell characters, such as the capacity of contact inhibition and the karyotype [9]. It has been shown that hTERT expression protects the WI38 transfected cells from stress-induced apoptosis and necrosis [10]. Prolonged culturing of WI38-hTERT cells leads to a loss of density-dependent growth inhibition and to an onset of contact-induced, p53 dependent cell death [11]. Our cell lines presented density-dependent growth inhibition. For the proteomics analysis, our three cell lines were maintained at 90% confluence, and in exponential growth phase. In these conditions, the population doubling levels were similar.

**Figure 1:**
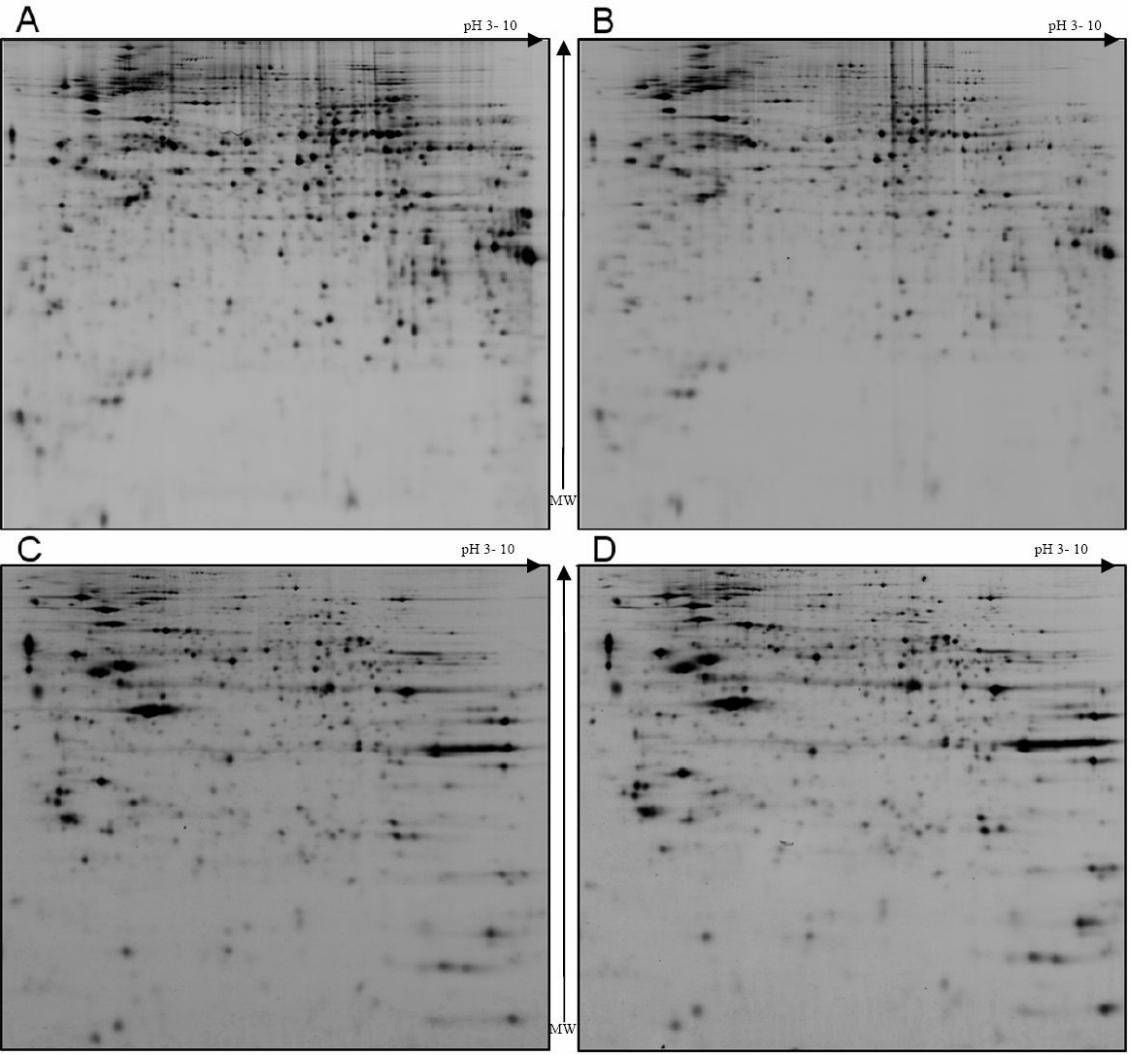
**Nuclear and cytosolic 2D DIGE gels**. Two dimensional gel electrophoresis images of nuclear (A, B) and cytosolic fractions (C, D) of WI38 cells. The subfractionation method used allows reproducible extraction and isolation of the nuclear and cytosolic sub-proteomes.

2D DIGE was performed on both nuclear and cytosolic cell fractions. The subfractionation method used relies on a complementary and very different 2D gel images (Figure 1) which is necessary for low abundant nuclear proteins differential analysis. Nuclear fraction corresponds to 5.89% ± 0.41 (mean value over 6 independent experiments) of the total protein content. Protein spots that showed either an increase or a decrease in intensity superior to 30%, together with a statistically significant Student's *t*-test (*p *< 0.05) were considered as being differentially expressed. Interestingly, numbers (Table 1) of differentially expressed proteins were found between parental cells and control HPV WI 38 cells. These modifications are thus a consequence of the general transfection and are not due to hTERT gene transfection. In this study only proteins which are differentially expressed between parental cells and hTERT cells and have in addition the same expression in parental cell and control HPV WI 38 cells were selected for the study and submitted to mass spectrometry based identification. In the cytosolic fraction, 57 spots out of 2246 are significantly differentially expressed (Table 1), and 38 were confidently identified (Table 2). In the nuclear fraction, 44 spots out of 2172 were selected in the differential proteome analysis (Table 1), and 14 were identified (Table 3).

**Table 1:** 2D DIGE spots selection.

	Nuclear fraction	Cytosolic fraction
Total number of spots differentially expressed in WI38/WI38-hTERT	190	210
Number of spots differentially expressed due to transfection (WI38/WI38-HPV)	146	153
By difference, number of spots differentially expressed due only to hTERT overexpression (number of identified spots are in parentheses)	44 (14)	57 (38)

Number of spots differentially expressed in 2D-DIGE experiment (Student's *t*-test *p *< 0.05 and increase or decrease intensity superior at 30%). Selection based on spots differentially expressed in WI38/hTERT WI 38, but similarly expressed in WI38 and control WI38-HPV.

**Table 2:** Cytosolic differentially expressed proteins identification and regulation

				**hTERTt-WI38/WI38**		**hTERT-WI38/HPV-WI38**		**Reference**
				
**Swiss-Prot AC**	**Gene name, STRING protein association network legend**	**Protein name**	**Function**	**T-test**	**Regulation**	**T-test**	**Regulation**	
[O43852]	CALR	Calumenin precursor	Ca(2+)-binding, secretory pathway	1.0E-03	2.27	1.6E-02	1.5	[40]
[Q15293]	RCN1	Reticulocalbin-1 precursor	Ca(2+)-binding, secretory pathway	1.3E-04	2.11	4.2E-04	1.85	[40]
[P14625]	HSP90B1	Endoplasmin precursor (GRP94)	ER chaperone, Ca(2+) binding, anti-apoptosis	1.4E-05	2.1	1.6E-03	1.26	[47, 59]
[Q14697]	GANAB	Neutral alpha-glucosidase AB precursor	Protein O-glucosyl hydrolase	4.9E-08	2.06	5.5E-06	1.42	
[O95302]	FKBP9L	FK506-binding protein 9 precursor (FKBP9)	Peptidyp propyl isomerase (PPI), accelerate protein folding	9.9E-05	2.04	3.9E-03	1.41	[52]
[Q9Y4L1]	HYOU1	150 kDa oxygen-regulated protein precursor (ORP150)	ER chaperone, exchange factor for GRP78	1.2E-03	1.98	2.3E-03	1.8	[47, 60]
[Q9BS26]	TXNDC4	Thioredoxin domain-containing protein 4 precursor (ERp44)	Control of oxidating protein folding, ERO1L partner	3.5^E^-06	1.89	7.2^E^-05	1.57	[61, 62]
[O43852]	CALU	Calumenin precursor	Ca(2+)-binding, secretory pathway	1.80E-05	1.80	3.7E-03	1.35	[40]
[Q15293]	RCN1	Reticulocalbin-1 precursor	Ca(2+)-binding, secretory pathway	2.5E-02	1.78	6.2E-02	1.51	[40]
[O75131]	CPNE3	Copine-3	Ca(2+)-dependent phospholipid-binding proteins	7.4E-04	1.73	1.2E-03	1.59	[63, 64]
[Q8NBS9]	TXNDC5	Thioredoxin domain-containing protein 5 precursor (Erp46)	Thiol oxydoreductase	3.2E-03	1.73	4.9E-03	1.65	[62, 65]
[P11021]	HSPA5	78 kDa glucose-regulated protein precursor (GRP78)	ER chaperone, Ca(2+)-binding, ER stress sensor, anti-apoptosis	1.5E-02	1.65	7.4E-03	1.57	[47, 66]
[P13674]	P4HA1	Prolyl 4-hydroxylase alpha-1 subunit precursor	Hypoxia-inducible collagen synthesis	2.0E-08	1.65	1.3E-06	1.77	[67]
[O15460]	P4HA2	Prolyl 4-hydroxylase alpha-2 subunit precursor	Hypoxia-inducible collagen synthesis	2.8E-04	1.64	1.5E-03	1.43	[67]
[P07237]	P4HB	Protein disulfide-isomerase precursor	Rearrangement of -S-S-bonds in proteins	2.0E-06	1.64	2.1E-04	1.28	[68]
[Q9H2D6]	TRIOBP	TRIO and F-actin-binding protein	May regulate actin cytoskeletal organization	8.0E-05	1.59	4.9E-03	1.35	
[P30101]	GRP58	Protein disulfide-isomerase A3 precursor (PDI-A3)	Rearrangement of -S-S-bonds in proteins	8.6E-07	1.57	2.8E-06	1.48	[68]
[Q96HE7]	ERO1L	ERO1-like protein alpha precursor (ERO1L)	Protein disulfide isomerases oxydoreductase	2.7E-02	1.54	4.7E-02	1.44	[61, 69]
[P27797]	CALR	Calreticulin precursor	ER chaperone, Ca(2+)-binding, glycoprotein folding	2.9E-02	1.46	7.8E-03	1.61	[47]
[Q15084]	PDIA6	Protein disulfide-isomerase A6 precursor (PDI-A6)	Rearrangement of -S-S-bonds in proteins	8.8E-05	1.45	1.1E-04	1.31	[68]
[Q99439]	CNN2	Calponin-2	Ca(2+)-binding, actin-binding RNA helicase which	2.1E-04	1.33	2.7E-03	1.25	[70, 71]
[P60842]	EIF4A1	Eukaryotic initiation factor 4A-I	mediates binding of mRNA to the ribosome	6.6E-05	1.33	8.6E-05	1.48	
[P30101]	GRP58	Protein disulfide-isomerase A3 precursor (PDI-A3)	Rearrangement of -S-S-bonds in proteins	3.9E-03	1.29	2.0E-03	1.44	[68]
[P27797]	CALR	Calreticulin precursor	ER chaperone, Ca(2+)-binding, glycoprotein folding	0.18	1.26	2.6E-02	1.68	[47]
[P51665]	PSMD7	26S proteasome non-ATPase regulatory subunit 7	Regulation of ubiquitinated proteins degradation	2.2E-02	0.76	1.4E-02	0.71	
[P04406]	HSD35	Glyceraldehyde-3-phosphate dehydrogenase	Glycolytic pathway	1.1E-02	0.75	7.7E-04	0.70	
[Q99426]	CKAP1	Tubulin-specific chaperone B	Chaperone, tubulin organisation	1.3E-03	0.75	8.8E-04	0.72	
[P04406]	HSD35	Glyceraldehyde-3-phosphate dehydrogenase	Glycolytic pathway	4.9E-03	0.74	9.4E-03	0.76	
[P09211]	GSTP1	Glutathione S-transferase P	Oxidative stress	6.1E-03	0.72	4.7E-02	0.81	
[P04075]	ALDOA	Fructose-bisphosphate aldolase A	Glycolytic pathway	6.4E-03	0.71	7.2E-02	0.85	
[P04075]	ALDOA	Fructose-bisphosphate aldolase A	Glycolytic pathway	8.2E-04	0.69	4.7E-02	0.75	
[P04406]	HSD35	Glyceraldehyde-3-phosphate dehydrogenase	Glycolytic pathway	1.2E-03	0.61	1.3E-03	0.69	
[P04792]	HSPB1	Heat-shock protein beta-1 (HSP27)	Chaperone, actin organization	3.8E-03	0.61	3.4E-03	0.65	
[P05120]	SERPINB2	Plasminogen activator inhibitor 2 precursor	Plasminogen activator inhibitor	3.3E-04	0.56	1.3E-03	0.59	
[P17987]	TCP1	T-complex protein 1 subunit alpha	Chaperone, protein folding, actin and tubulin folding	1.7E-02	0.56	3.7E-03	0.67	
[P08758]	ANXA5	Annexin A5	Ca(2+)-dependant phospholipids-binding, apoptosis regulation	2.1E-04	0.39	2.6E-04	0.40	
[P07737]	PFN1	Profilin-1	Intranuclear movements, assembly of transcription complexes	3.3E-04	0.38	1.0E-02	0.33	
[P07737]	PFN1	Profilin-1	Intranuclear movements, assembly of transcription complexes	3.2E-02	0.37	2.7E-02	0.4	

List of the proteins differentially expressed due to hTERT transfection in the cytosolic fraction of human WI38 cells. Listed by decreasing regulation.

**Table 3:** Nuclear differentially expressed proteins identification and regulation

				**hTERTt-WI38/WI38**		**hTERT-WI38/HPV-WI38**	
				
**Swiss-Prot AC**	**Gene name, STRING protein association network legend**	**Protein name**	**Function**	**T-test**	**Regulation**	**T-test**	**Regulation**
[P31943]	HNRPH1	Heterogeneous nuclear ribonucleoprotein H	Pre-mRNAs processing	4.4E-03	1.73	3.0E-02	1.58
[P08235]	NR3C2	Mineralocorticoid receptor	Binds to mineralocorticoid response elements (MRE) and transactivates target genes	6.1E-03	1.65	1.6E-02	1.53
[O14579]	COPE	Coatomer subunit epsilon	Implicated in vesicles trafficking	4.2E-02	1.62	0.12	1.33
[Q05048]	CSTF1	Cleavage stimulation factor 50 kDa subunit	Polyadenylation and 3'-end cleavage of mammalian pre-mRNAs	1.5E-03	1.57	5.6E-03	1.64
[P07900]	HSPCA	Heat shock protein HSP 90-alpha	Chaperone, 680kDa human telomerase complex partner [72]	1.5E-02	1.42	2.2E-04	2.05
[P62826]	ARA24	GTP-binding nuclear protein Ran	nucleocytoplasmic transport	2.0E-02	1.37	6.7E-03	1.24
[O43809]	ENSP0000030 0291	Cleavage and polyadenylation specificity factor 5	Polyadenylation and 3'-end cleavage of mammalian pre-mRNAs	1.6E-02	1.35	-	1.35
[Q13838]	ATP6V1G2	splicesome RNA helicase BAT1	Splice factor, required for mRNA export	6.3E-03	1.34	1.7E-03	1.5
[P25788]	PSMA3	Proteasome subunit alpha type 3	Protein degradation	5.6E-03	0.68	4.7E-02	0.75
[P28072]	PSMB6	Proteasome subunit beta type 6 precursor	Protein degradation	3.8E-05	0.65	3.0E-04	0.66
[P08865]	RPSA	40S ribosomal protein SA	Protein synthesis	1.3E-04	0.58	5.4E-04	0.60
[P60842]	EIF4A1	Eukaryotic initiation factor 4A-I	RNA helicase which mediates binding of mRNA to the ribosome	3.1E-02	0.54	1.2E-01	0.52
[P09382]	LGALS1	Galectin-1	Cell growth, apoptosis and cell differentiation	8.8E-07	0.50	1.2E-06	0.51
[Q12906]	ILF3	Interleukin enhancer-binding factor 3	Translation inhibitory protein of acid beta-glucosidase and other mRNAs, IL2 transcription regulator, promote the formation of stable DNA-dependent protein kinase holoenzyme complexes on DNA	1.1E-05	0.47	9.5E-04	0.66

List of the proteins differentially expressed due to hTERT transfection in the nuclear fraction of human WI38 cells. Listed by decreasing regulation.

### Functional protein association network of the proteins differentially expressed due to hTERT transfection [12]

**Figure 2:**
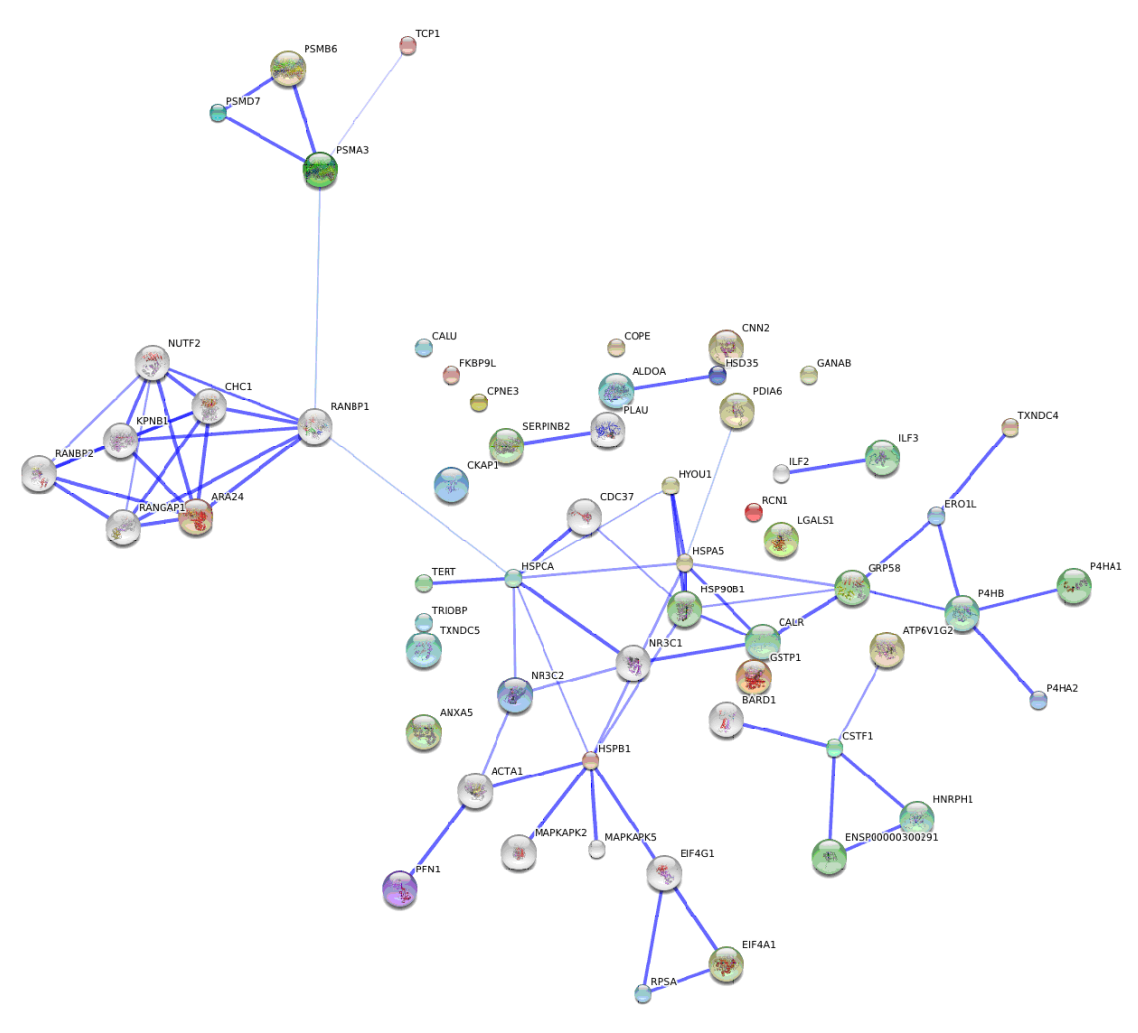
**Protein association network of the proteins differentially expressed due to hTERT transfection**. Protein-protein interactions predictions from the identified proteins which have their expression modified due to hTERT transfection. Based on STRING [12]. Network Display – Nodes are either coloured (differentially expressed proteins in this study except TERT that was manually introduced) or white (nodes of a higher iteration/depth). Additional white nodes are automatically generated by STRING to maximize existing interactions. Stronger associations are represented by thicker lines. Parameters used to create the protein association network: medium confidence and 15 additional (white) nodes. Protein descriptions of the coloured nodes are described in Table 2 and 3 those of white nodes in Table 4.

Based on STRING protein-protein interactions predictions an association network of the proteins which have their expression modified due to hTERT transfection was created (Figure 2). Table 4 describes the 15 proteins that were automatically selected by STRING to enlarge the protein association network. The bioinformatics analysis evidenced protein groups which have their expression modified due to hTERT transfection in which proteins involved in ubiquitin-proteasome protein degradation pathway, nuclear-cytosol transport, endoplasmic reticulum functions, pre-mRNAs processing and cell several chaperone functions. This network will be further compared to those of anti telomerase drugs experiments.         

**Table 4:** Protein descriptions of the white nodes of the Protein association network (Figure 2)

**Gene name, STRING protein association network legend**	**Protein name**
**ACTA1**	Actin, alpha skeletal muscle (Alpha-actin-1)
**BARD1**	BRCA1-associated RING domain protein 1 (BARD-1)
**CDC37**	Hsp90 co-chaperone Cdc37 (Hsp90 chaperone protein kinase-targeting subunit) (p50Cdc37)
**CHC1**	Regulator of chromosome condensation (Cell cycle regulatory protein)
**EIF4G1**	Eukaryotic translation initiation factor 4 gamma 1 (eIF-4-gamma 1) (eIF-4G1) (eIF-4G 1) (p220)
**ILF2**	Interleukin enhancer-binding factor 2 (Nuclear factor of activated T-cells 45 kDa)
**KPNB1**	Importin beta-1 subunit (Karyopherin beta-1 subunit) (Nuclear factor P97) (Importin 90)
**MAPKAPK2**	MAP kinase-activated protein kinase 2 (EC 2.7.1.-) (MAPK-activated protein kinase 2) (MAPKAP kinase 2) (MAPKAPK-2)
**MAPKAPK5**	MAP kinase-activated protein kinase 5 (EC 2.7.1.37) (MAPK-activated protein kinase 5) (MAPKAP kinase 5) (p38-regulated/activated protein kinase)
**NR3C1**	Glucocorticoid receptor (GR)
**NUTF2**	Nuclear transport factor 2 (NTF-2) (Placental protein 15) (PP15)
**PLAU**	Urokinase-type plasminogen activator precursor (EC 3.4.21.73) (uPA) (U-plasminogen activator) [Contains: Urokinase-type plasminogen activator long chain A; Urokinase-type plasminogen activator short chain A; Urokinase-type plasminogen activator chain B]
**RANBP1**	Ran-specific GTPase-activating protein (Ran binding protein 1) (RanBP1)
**RANBP2**	Ran-binding protein 2 (RanBP2) (Nuclear pore complex protein Nup358) (Nucleoporin Nup358) (358 kDa nucleoporin) (P270)
**RANGAP1**	Ran GTPase-activating protein 1

### Up regulation of the heat shock protein 90-alpha

The heat shock protein 90-alpha (hsp90α) is upregulated by a factor 1.42 in the nuclear fraction of hTERT WI38 cells. Hsp90α exerts its chaperon function to ensure the correct conformation, activity, intracellular localization and proteolytic turnover of a range of proteins that are involved in cell growth, differentiation and survival [13]. More specifically, hsp90α interacts with telomerase complex and occupy a central place in our protein association network (Figure 2). Hsp90α is involved in allowing RNA template of the telomerase (hTR) to bind hTERT and also in fine tuning and stabilizing the structure of the telomerase complex [14,15]. Its central role in the telomerase activity modulation and in multiple signaling pathways and biological processes makes it a relevant target for anti cancer therapy. Number of study are currently evaluating clinical activity of hsp90 inhibitors in cancer therapeutics [16-22]. Our DIGE study links the overexpression of the hsp90α with the ectopic expression of hTERT and suggests a key role of this chaperone in the subsequent cell adaptation to immortalization. In addition, Hiyama and coworkers have shown in a differential gene expression profiles study that the hsp90α is overexpressed in tumor with high telomerase activity comparing tumor with low telomerase activity [23]. Theses findings emphasize the interest of anti hsp90 targeting drugs therapy and shows the interest of such proteomics methodology for potential biomarkers discovery and further drugs candidates characterization and preclinical validation.

### Down-regulation of apoptotic effectors Galectin-1 and Annexin 5

Galectin-1 (Gal-1) is downregulated by a factor of 2.1 in the nuclear proteome of hTERT-transfected cells. Gal-1 is a highly conserved protein with a carbohydrate-recognition domain that binds beta-galactoside. Recently Gal-1 was shown to be implicated in cell growth, apoptosis and cell differentiation and survival of effector T cells [24-27]. Exogenously added recombinant Gal-1 induces apoptosis by the mitochondrial and death receptor pathways of MA-10 tumor Leydig cells. In contrast, low concentrations of Gal-1 significantly promote cell proliferation, without inducing cell death [26]. Walzel and al. have shown that Gal-1 triggers through binding to N-linked glycans a Ca^2+- ^sensitive apoptotic pathway [28].

Annexin 5 is downregulated by a factor of 2.64 in the cytosolic proteome of hTERT-transfected cells. Annexins are abundant Ca^2+- ^dependant phospholipids-binding proteins. Depletion of endogenous annexin 5 with siRNA inhibits delta protein kinase C functions in which cellular processes such as growth, differentiation and apoptosis [29,30]. In addition Hawkins and co-workers have proven that annexin 5 lack expression results in reduced susceptibility to a range of apoptotic stimuli, and that annexin 5 (-/-) cells are more resistant to apoptosis [31].

The downregulation of these two proteins in hTERT-transfected cells suggests that telomerase expression, in addition to its immortalization effect, induces a protection against apoptosis, compared to WI 38 cells. In agreement, modulation of apoptosis have been reported [32-34].

### Upregulation of Cajal bodies associated factors

Cleavage stimulator factor 50 kDa subunit (CstF-50) is upregulated by a factor of 1.57 (T-test: 1.5E-03), and cleavage and polyadenylation specificity factor 5 (CPSF-25) by a factor of 1.35 (T-test: 1.6E-02) in the nuclear proteome of hTERT transfected cells. CstF-50 is a subunit of the heterotrimer CstF. CstF and CPSF-25 are required for polyadenylation and 3'-end cleavage of mammalian pre-mRNAs. Schul and co-workers have shown that "cleavage bodies" (compact spherical fibrous structures containing CstF et CPSF factors) are intimately associated with Cajal bodies (CBs) [35]. hTERT and the telomerase RNA component, hTR, are also found in CBs. Ectopic expression of hTERT results in accumulation of hTR in CBs in primary fibroblasts or smooth muscle cells, but not in normal cells or in telomerase-negative (ALT) tumor cells [36]. It has been proposed that CBs could act as storage sites and deliver components of the telomerase complex when needed [37]. hTR localization in CBs is an important regulatory mechanism for telomere length homeostasis in human cells: mutant hTR failing to accumulate in CBs results in a functional deficiency and a decreasing association of telomerase with telomere [38]. The RNA component of human telomerase (hTR) includes H/ACA and CR7 domains required for 3' end processing, localization and accumulation. 3' end processing is a prerequisite for translocation of hTR to CBs [38]. A probable role of CstF-50 and CPSF-25 in hTR maturation may be an important regulation process and requires further investigations.

Profilin 1 is a known component of Cajal bodies supposed involved in both ATP-dependant intranuclear movements and in the assembly of transcription complexes [39]. Our results show that this protein is downregulated in cytosol hTERT transfected cells. Modulation of this protein may also implicate CBs functions in hTERT-transfected cells.

### Enhanced protective ER functions in hTERT transfected cells

22 out of the 38 proteins identified in the cytosolic fraction are involved in endoplasmic reticulum functions and are up-regulated in the hTERT transfected cells. Our results are consistent with this recent report and underline the implication of the CERC protein family as an important signaling pathway in hTERT transfected cells. Reticulocalbin and calumenin belong to the CREC (Cab45, reticulocalbin, ERC-45, calumenin) proteins, which constitute a family of EF-hand (helix-turn-helix structural motif) calcium binding proteins localized to the secretory pathway [40]. It has been proposed that CREC family is essential for cell survival because homozygous deletion of a region containing the reticulocalbin gene is lethal [41]. Several reports described their important role in pathophysiological processes especially in connection with malignant transformation [42-44]. Another recent study indicates that calumenin may have an autocrine or a paracrine effect on the cells in its vicinity modulating cell cycle and organization of the actin cytoskeleton [45]. Additionally, calumenin and reticunocalbin were also differentially expressed in a study comparing early passage, senescent, and hTERT-transfected endothelial cells [46]. In this study, mRNA level of these proteins were upregulated in hTERT transfected cells compared to early passage subconfluent cells.

ORP150, GRP78, calreticulin, PDIs, ERp44, ERp46, FKBP9 are implicated in essential ER functions and Ca^2+ ^homeostasis [47]. Interestingly, gene knockout experiments have proven that ER chaperon function is required for early mammalian development. Depletion of GRP78 leads to lethality in 3.5-day-old embryos (E3.5) due to failure of embryo peri-implantation [48]. This study suggests that physiological ER stress may exist in early development due to increased activity of cell proliferation and protein secretion. Calreticulin deficiency is also lethal in mouse embryos at E14.5, resulting from a lesion in cardiac development [49]. Interestingly GRP78 and calreticulin are downregulated after birth in the healthy mature heart [50]. ER chaperones overexpression are also proposed to promote cancer and tumor immunity [47]. It is interesting to note that telomerase is generally active in early embryonic stage and in 90% of cancers like these chaperones.

Our results indicate that overexpression of ER chaperones is a consequence of telomerase reactivation. The fact that these proteins are selectively upregulated in hTERT WI38 cells compared to WI38 and WI38-HPV control cells demonstrates that hTERT induces an important modulation of the ER functions. The ER is one of the most important folding compartments within the cell, as well as an intracellular Ca^2+ ^storage organelle and it contains a number of Ca^2+ ^regulated molecular chaperones responsible for the proper folding of glycosylated as well as non-glycosylated proteins. ER is also capable, through Ca^2+ ^homeostasis modulation, to determine cellular sensitivity to ER stress and apoptosis [51]. We propose that upregulations of ER chaperones in hTERT transfected cells are responsible of a protective cell effect essential for the proliferation of hTERT immortalized cells. This protective effect is probably mediated by upregulation of GRP78 and calreticulin chaperones. In addition, FKBP9 may play an important role in the control of the timing of this biological process due to the recent discovery of its molecular timer function [52]. We propose also that intracellular and potent intercellular anti-apoptotic signaling are mediated by proteins belonging to the CREC family in hTERT positive cells trough a Ca^2+ ^signaling modulation. Such proteome modulation makes evidence that telomerase reactivation, in addition of elongating telomere, has several indirect effects that augment ER capacity of protein folding and degradation [53-55].

## Conclusion

We show that the methodology reduces the complexity of the proteome analysis and highlights proteins implicated in other processes than telomere elongation. hTERT transfection enhances natural ER capacity and modulates Ca^2+ ^cell signaling pathways potentially resulting in overprotection mechanisms against endogeneous and exogeneous disorder. This hypothesis is in accordance with the identified down-regulation of apoptotic effectors Galectin-1 and Annexin 5. Other effects like an enhanced DNA excision repair pathway have also been reported in cell with long telomere [56]. Altogether, these observations suggest that telomerase expression enhances natural cell repair mechanisms and stress resistance probably required for long term resistance of immortalized cells. Therefore, hTERT transfected cells cannot be only considered as an immortal equivalent to parental cells but also as cells which are over-resistant to stresses. The introduction of hTERT gene in WI38 cells modulates the chaperone hsp90α expression which seems to be, by its central position in the protein association network (Figure 2), an important regulator of subsequent cell adaptation mechanism to immortalization. This finding increases the interest of current anti cancer studies based on hsp90 inhibition. In addition to these observations, orthogonal analysis of our results with several cancerous cells proteomic studies reveals that some of the current highlighted proteins such as hsp90α, GRP78 and calreticulin are also implicated in oncogenese and cell resistance [53-55]. Finally the model "parental WI38/hTERT WI38/HPV WI38" characterization (Table 2, 3) is the prerequisite for any larger proteomics aiming to evaluate anti-telomerase drugs proteome alteration and thus therapeutics induced cell reactions.

## Methods

### Cell culture and infection of WI38 cells

hTERT WI38, HPV WI38 and parental WI38 cells (human embryonic lung fibroblasts) were grown in Eagle's minimal essential medium with Glutamax (Invitrogen), supplemented with 10% fetal calf serum and penicillin-streptomycin 1% (Gibco).

Lentiviral supernatants containing hTERT or control HPV vector were a generous gift from Dr. Annelise Bennaceur-Griscelli (Institut Gustave Roussy, Villejuif, France). Briefly, WI 38 cells at 1.5 × 10^5 ^cells/mL were infected at a multiplicity of infection equal to 50 in the presence of 4 μg/mL Polybrene in complete culture medium. Enhanced green fluorescent protein-positive cells were sorted 5 days later by flow cytometry according to a high or low intensity of fluorescence. Populations that expressed a high intensity of fluorescence were seeded [57].

### Non denaturing subfractionation method

The non denaturing method of extraction and fractionation is based on the method of Gorski and co-workers [58] and has been adapted for WI38 cells. A cytosolic fraction and a nuclear histone-depleted proteins fraction were obtained. We verified the presence or not of telomerase activity by Western blots and TRAP (telomeric repeat amplification protocol) assay on each fraction. Then, a 2D LC-MS/MS analysis was performed and allowed the identification of more than 100 proteins per fraction. The two fractions shared a few number of common structural proteins and the nuclear fraction was enriched in low abundant proteins such as transcription factors and telomeric proteins (results not shown). For each experiment, 25 T 175 flasks 90% confluent are rinsed three times with PBS, then collected, using a scraper in a cold room at 4°C. The collected cells are centrifuged 5 minutes at 400 g and recovered. 10 mL of buffer 1 (Hepes-KOH 10 mM, pH 7.6; KCl 10 mM; Spermine/HCl 0.15 mM; Spermidine 0.5 mM; DTT 0.5 mM) (10 times a volume equivalent to the fresh weight) is added to the cell pellet, homogenized and incubated 10 minutes on ice. The tube is then centrifuged 5 minutes at 800 g and the cells are recovered. One volume equivalent to the fresh weight of buffer 1 is added. The solution is homogenized with 10 strokes with a motorized potter Elvehjem homogenizer (Teflon/glass) at 4000 rpm and the disruption of the cell membrane is controlled by Trypan Blue staining of nuclei with an inverted phase contrast microscope. 10% in volume of buffer 2 (Hepes-KOH 10 mM, pH 7.6; KCl 1 M; Spermine/HCl 0.15 mM; Spermidine 0.5 mM; DTT 0.5 mM) is added to restore isotonicity of the solution, the solution is homogenized with 10 strokes at 4000 rpm and centrifuged 10 minutes at 1100 g. The pellet which contains nuclei is recovered, the supernatant is collected and centrifuged 30 minutes at 24000 g. The supernatant is recovered and corresponds to the cytosolic fraction. The pellet (which contains nuclei) is homogenized in buffer 4 (Hepes-KOH 10 mM, pH 7.6; KCl 100 mM; Spermine/HCl 0.15 mM; Spermidine 0.5 mM; DTT 0.5 mM) with 5 strokes at 800 rpm and centrifuged 5 minutes at 1100 g. This step is repeated 3 times. The cleaned nuclei are homogenized in 2.25 mL of buffer (Hepes-KOH 10 mM, pH 7.6; KCl 100 mM; DTT 0.5 mM) with 5 strokes at 800 rpm and transferred to a 3 mL ultra-centrifuge tube. 250 μL of ammonium (10% V/V) sulfate is gently added and slowly agitated for 30 minutes. Then, a centrifugation is done at 90000 g for 40 minutes at 4°C. The supernatant which contains nuclear proteins is transferred to another tube. 0.3 g/mL of ammonium sulfate are added to the solution and let under smooth agitation for 40 minutes. The solution is centrifuged at 90000 g for 30 minutes at 4°C. 1 mL of buffer 7 (Hepes-KOH pH 7.6 25 mM; KCl 150 mM; DTT 1 mM) is added to the supernatant after the centrifugation and shaken for 15 minutes. The solution is dialyzed overnight and 2 hours again in buffer 7. The solution is centrifuged at 24000 g for 5 minutes. The supernatant is collected and constitutes the nuclear fraction.

### CyDye Labeling and Two-Dimensional Differential In-Gel Electrophoresis (2D-DIGE)

DIGE technology was used with at least triplicate experiment for each nuclear and cytosol subproteomes from WI38, hTERT-WI38 and HPV-WI38. The DIGE technology allows 3 different protein fluorescent labelling by the use of Cy dyes. Two different samples are labelled by Cy3 and Cy5, whereas a pool of all samples is labelled by Cy2. A total of 12 2D-DIGE gels were realised corresponding to 24 different samples applied. The Cy2 internal standard sample reduces inter-gel experimental variations resulting in statistically improved results. Protein samples (12.5 μg each at 5 mg/mL) were labelled in 7 M urea, 2 M thiourea, 1.5% (w/v) ASB-14, 1.5% (w/v) CHAPS, 20 mM TRIS-HCl, pH 8.5, according to the manufacturer procedure (Amersham Biosciences part of GE Healthcare). Samples were applied at least in triplicate with inversing Cy3/Cy5 labelling. An internal standard constituted by a mix of all samples was Cy2 labelled. Differentially labelled samples (12.5 μg of each Cy2-, Cy3-, and Cy5-labeled sample) were pooled and resolved isoelectrically on 24-cm IPG strips, pH 3–10, NL on a Protean IEF cell (Bio-Rad). After active rehydration for 9 h with the sample, the isoelectric focalisation is carried out up to 65000 Vh over night. IPG strips were seeded in reduction buffer for 15 minutes (DTT 130 mM, urea 6 M, Tris-HCl 0.373 M, pH 8.8, glycerol 20% v/v, SDS 2% w/v) followed by an alkylation (iodoacetamide 135 mM, urea 6 M, Tris-HCl pH 8.8 0.373M, glycerol 20% v/v, SDS 2% w/v) for additional 15 minutes. The second dimension electrophoresis was performed overnight at 20°C in an Ettan Dalt II system (G.E. Healthcare) at 1 W per gel. Each gel was finally scanned with the Typhoon 9400 scanner (G.E. Healthcare) at the wavelengths corresponding to each CyDye. Images were analyzed with the DeCyder software 6.5 (G.E. Healthcare) according to the manufacturer. Protein spots that showed a statistically significant Student's *t*-test (*p *< 0.05) for an increased or decreased in intensity superior at 30% were accepted as being differentially expressed. Only spots that had similar abundances in WI38 and WI38-HPV, and were differentially expressed in WI38-hTERT were selected for MS identification.

### Protein identification

**Figure 3:**
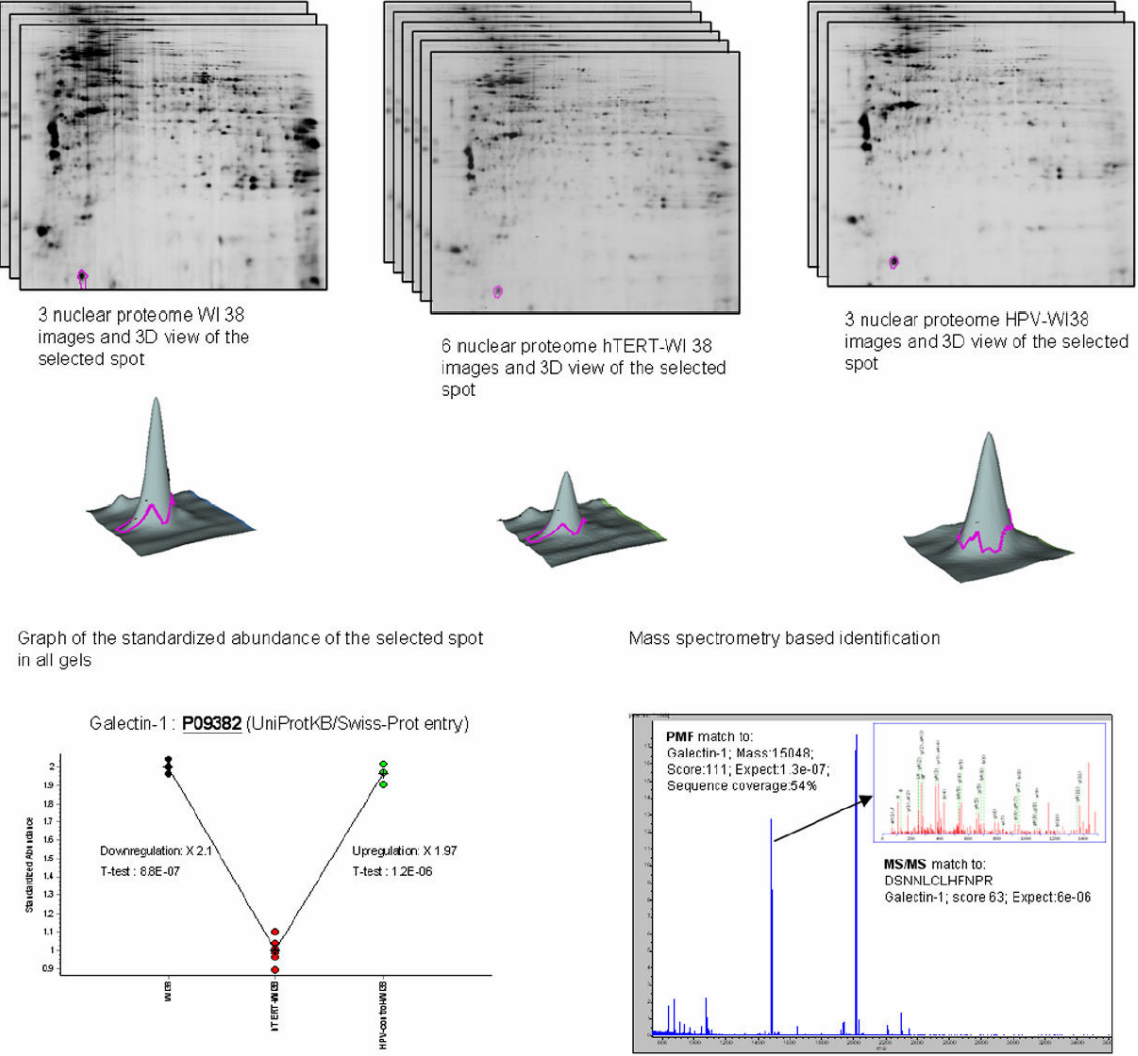
**Experimental design**. Figure 3 shows an example of the experimental design applied for differential 2D-DIGE analysis and mass spectrometry based identification. Nuclear sub-proteomes from WI38, hTERT WI38 and HPV-WI38. Each spot is selected from the standardized abundance, then excised and digested for MS/MS analysis.

Spots of interest were automatically excised from the gel with the Ettan Spot Picker and submitted to tryptic digestion. The resulting peptides were extracted and rehydrated in 10 μL of formic acid (1%). One microliter of sample was deposited on an Ancorchip MALDI plate prespotted with HCCA matrix (Bruker-Daltonics). Samples were analyzed with an UltraFlex II MALDI-TOF-TOF (Bruker Daltonics) by MS fingerprint (spectra acquisition mass range:70–4000 *m*/*z*). Peaks with the highest intensities obtained in TOF-MS mode were subsequently analyzed by LIFT MS/MS for confirmation (mass range 40–4000). Protein identifications were carried out using the Biotools software (version: 3.0, build 2.9, Bruker Daltonics), the Mascot search engine (Version: 2.1.0) and SwissProt database (Sprot 50.8). All spots discussed here had a score corresponding to a p-value < 0.001 (where P is the probability that the observed match is a random event). Identification with a p-value between 0.001 and 0.05 were all manually confirmed. Figure 3 shows an example of the general procedure applied for differential 2D analysis and mass spectrometry based identification.

## Competing interests

The authors declare that they have no competing interests.

## Authors' contributions

GDM, EDP, MC DPG, JFR, VG designed the study and interpreted part of the results. GDM performed most of the experiments, analyzed most of the results and drafted the manuscript. FR performed part of bioinformatics setup for mass spectrometry based identification. NS, MF and WA performed part of sample preparation and 2D DIGE experiments. All authors read and approved the final manuscript.
